# Utility of Genetic Testing for Confirmation of Abnormal Newborn Screening in Disorders of Long-Chain Fatty Acids: A Missed Case of Carnitine Palmitoyltransferase 1A (CPT1A) Deficiency

**DOI:** 10.3390/ijns3020010

**Published:** 2017-04-28

**Authors:** Leah Dowsett, Lauren Lulis, Can Ficicioglu, Sanmati Cuddapah

**Affiliations:** 1 Department of Pediatrics, Division of Human Genetics, The Children’s Hospital of Philadelphia, 3401 Civic Center Boulevard, Philadelphia, 19104 PA, USA; 2 Perelman School of Medicine at the University of Pennsylvania, Philadelphia, 19104 PA, USA

**Keywords:** carnitine palmitoyltransferase deficiency, CPT1A, fatty acid oxidation disorders, elevated liver transaminases, Ashkenazi Jewish, neonatal screening

## Abstract

An 18-month-old male was evaluated after presenting with disproportionately elevated liver transaminases in the setting of acute gastroenteritis. He had marked hepatomegaly on physical exam that was later confirmed with an abdominal ultrasound. Given this clinical picture, suspicion for a fatty acid oxidation disorder was raised. Further investigation revealed that his initial newborn screen was positive for carnitine palmitoyltransferase 1A (CPT1A) deficiency—a rare autosomal recessive disorder of long-chain fatty acid oxidation. Confirmatory biochemical testing in the newborn period showed carnitine levels to be unexpectedly low with a normal acylcarnitine profile. Thus, it was considered to be a false-positive newborn screen and metabolic follow-up was not recommended. Repeat biochemical testing during this hospitalization revealed a normal acylcarnitine profile. The only abnormalities noted were a low proportion of acylcarnitine species from plasma, an elevated free-to-total carnitine ratio, and mild hypoketotic medium chain dicarboxylic aciduria on urine organic acids. Gene sequencing of CPT1A revealed a novel homozygous splice site variant that confirmed his diagnosis. CPT1A deficiency has a population founder effect in the Inuit and other Arctic groups, but has not been previously reported in persons of Ashkenazi Jewish ancestry.

## 1. Introduction

Carnitine palmitoyltransferase 1A (CPT1A) deficiency is a rare autosomal recessive disorder of long-chain fatty acid oxidation. Patients are at risk for hypoglycemia and liver failure during times of fasting or illness. Lab abnormalities seen during such a crisis includes hypoglycemia, hyperammonemia, low acylcarnitines, elevated levels of total and/or free carnitine, transaminitis, and elevated coagulation studies. Diagnosis is established by corresponding biochemical profile and confirmed with molecular sequencing of the CPT1A gene. Residual enzyme activity is typically reduced to 1%–5% in affected individuals, and testing for this via skin fibroblasts is recommended when molecular sequencing is unavailable or inconclusive. The mainstay of treatment is dextrose-containing IV fluids to replete hepatic glycogen stores to prevent metabolic crisis and hepatic encephalopathy [1].

Here, we describe a patient who had normal biochemical profiles in the newborn period who was subsequently diagnosed with CPT1A deficiency only after molecular testing revealed him to be homozygous for a splice-site variant in this gene. This condition, which has a population founder effect in the Inuit and other arctic groups, has not been previously reported in persons of Ashkenazi Jewish ancestry [2–4].

## 2. Case Report

An 18-month-old male child presented complaints of elevated liver transaminases in the form of vomiting and diarrhea. He was initially presented to a local hospital where he was admitted for treatment of dehydration secondary to acute viral gastroenteritis. On the second day of hospitalization, his condition worsened and he was transferred to our facility for further evaluation and management. His initial labs revealed normal CK, ammonia, and coagulation studies. However, he did have an elevated AST (517 U/L, normal 20–60 U/L) and ALT (388 U/L, normal 5–25 U/L). Alkaline phosphatase was also normal (191 U/L, normal 145–320 U/L). On further investigation, hepatic function testing revealed elevated liver transaminases that were nearly 10-fold elevated from normal values. Creatine phosphokinase (CK) was at the upper end of normal (304 U/L, normal 60–305 U/L). Ultrasound of the abdomen revealed an enlarged liver with increased heterogeneous echogenicity and a thickened gallbladder wall with slightly complicated appearing ascites.

Given this clinical picture, suspicion for a fatty acid oxidation disorder was raised, and the metabolism team was consulted for evaluation. Further history obtained from the family revealed that the patient was the fourth child born to non-consanguineous parents of Ashkenazi Jewish ancestry. He was born at term and there were no complications reported during the pregnancy. There was also no history for teratogenic exposures during the pregnancy. Parents reported that everything in the newborn period was routine. This patient had no prior history of documented hypoglycemia. He had both motor and speech delays, and at the time of presentation at 18 months of age had just begun to walk and knew one or two words. His weight was 10.9 kg (24%), length was 83.0 cm (61%), and weight-for-length was at the 25th percentile. Physical examination at the time revealed mild dysmorphic features including a beaked nose and mild retrognathia. Abdominal exam was notable for hepatomegaly 4 cm below the right costal margin. The child also had truncal hypotonia.

**Figure 1:**
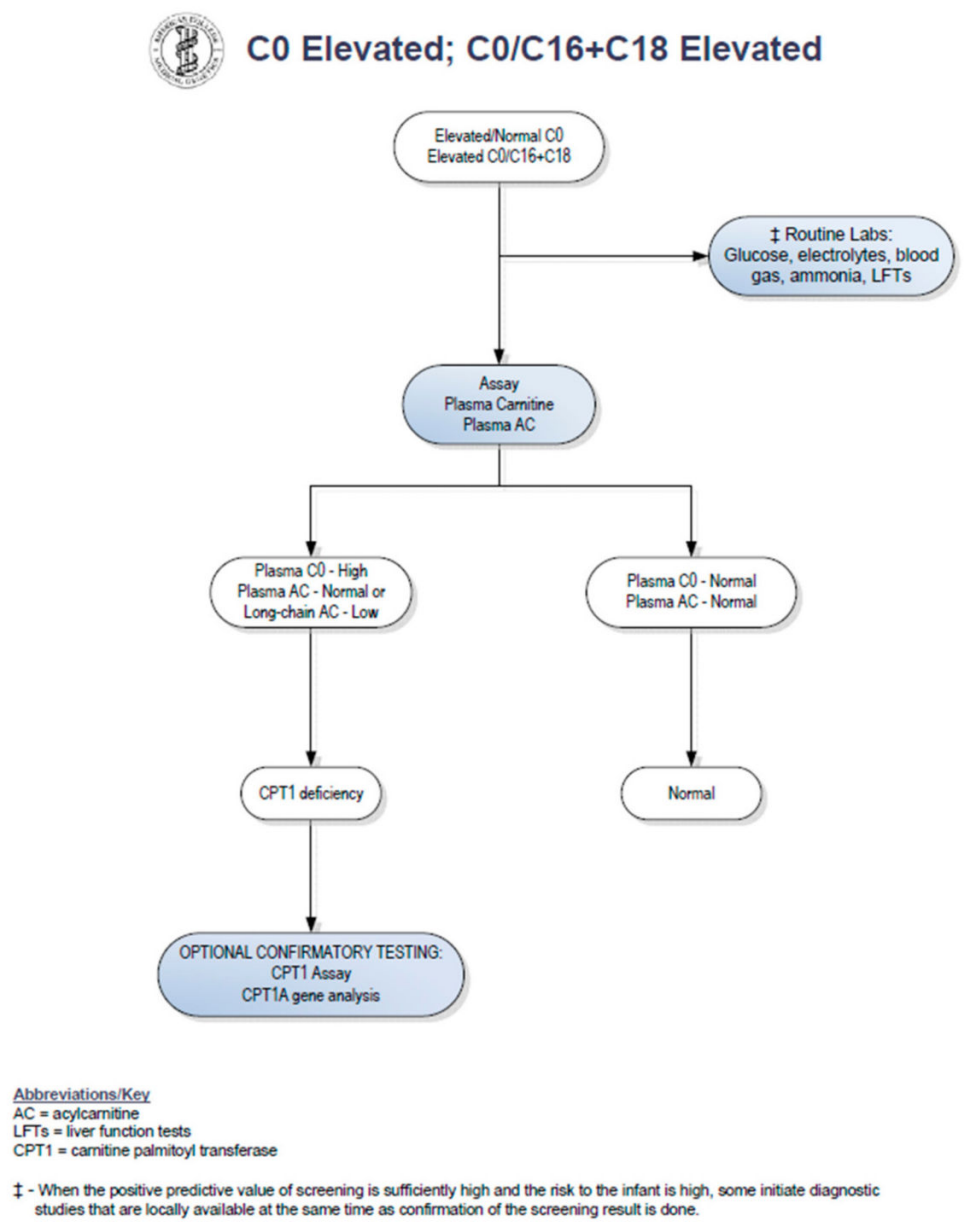
Confirmatory algorithm for CPT1 deficiency on newborn screening. Reprinted with permission from [7]. Copyright 2006, American College of Medical Genetics and Genomics.

Upon review of the patient’s newborn screen, it was found that his screen had been positive for CPT1 deficiency at birth. A positive newborn screen results when there is a low C16 value with increased ratios of free carnitine to the sum of C16:0 plus C18 acylcarnitines [5]. The C16 was low at 0.21 umol/L (normal > 0.66 umol/L). The C0 was normal at 80.44 (cutoff < 137), and the C0/C16 + 18 ratio was 278.75. Records indicated that his primary care physician sent an acylcarnitine profile and carnitine levels from plasma based on recommendations from a metabolic physician. Results of these studies showed carnitine levels to be unexpectedly low. Total carnitine was 16 mmol/L (normal 33–70 mmol/L) and free carnitine was 14 mmol/L (normal 28–52 mmol/L). The acylcarnitine profile was normal. In a study by de Sain-van der Velden et al., it was demonstrated that CPT1A deficiency can be missed when analysis is performed on plasma alone. Because long-chain acylcarnitines are absorbed on the surface of red blood cells, dried blood spots are the preferred sample type for diagnostic analysis [6]. No repeat newborn screen was sent. Given these findings, the case was considered to be a false-positive result (see Figure 1) and no metabolic follow-up was recommended by his providers at that time.

With the patient’s prolonged elevated liver transaminases during the current hospitalization and history of an abnormal newborn screen, the diagnosis of CPT1A was again considered during his hospitalization at 18 months of age. Aside from a positive newborn screen, other expected supportive laboratory findings in patients with CPT1A deficiency include hypoketotic hypoglycemia, elevated AST/ALT levels (2–10 times the upper limit of normal), hyperammonemia, elevated total serum carnitine, and elevated C6–12 species on urine organic acids [8]. Our patient did not have documented hypoglycemia during his hospital course. He had normal total serum carnitine but slightly elevated free carnitine levels (total carnitine 67.8 nmol/mL, normal 25–69 nmol/mL; free carnitine 62.6 nmol/mL, normal 17–59 nmol/mL; acylcarnitine 5.2 nmol/mL, normal 4–14 nmol/mL). His C16 and C18 from plasma were 0.03 and 0.01 umol/L, respectively. His ammonia level was normal (< 9 mmol/L). Though the values were reported as normal, closer review of his laboratory studies showed that the acylcarnitine species only comprised 8% of the sample, whereas normal patients without a disorder of fatty acid oxidation are expected to have > 20% acylcarnitine species present in their plasma. His free-to-total carnitine ratio was elevated at 0.92 (normal 0.68–0.86), also suggestive of CPT1A deficiency. His acylcarnitine profile was again within normal limits. Aside from the elevated liver transaminases, the only additional biochemical evidence to suggest CPT1A deficiency was the presence of hypoketotic medium chain dicarboxylic aciduria on urine organic acids.

Gene sequencing of CPT1A revealed the patient to be homozygous for a splice site variant that destroys the canonical splice donor site in intron 10 (c.1163 + 1 G > A) and was therefore reported as pathogenic. This variant has not been previously reported.

In outpatient follow-up one month later, the AST and ALT had normalized and his other biochemical labs remained unremarkable. He was started on a low-fat diet with medium chain triglyceride supplementation. The nature of the condition was discussed with the family including avoidance of fasting and dietary management. The recurrence risk for future pregnancies was discussed, and testing for family members (parents and siblings) was encouraged. The patient was seen again for routine follow-up in a metabolism clinic six months later. He had not had any intercurrent illness or evidence of metabolic decompensation. The family reported that his energy level improved after starting medium chain triglyceride supplementation. Again, genetic counseling was provided, and testing of siblings was recommended given the possibility of one of the siblings having a mild form of disease that could have also been missed on newborn screening. Genetic testing confirmed that none of the other siblings were affected.

## 3. Discussion

**Figure 2:**
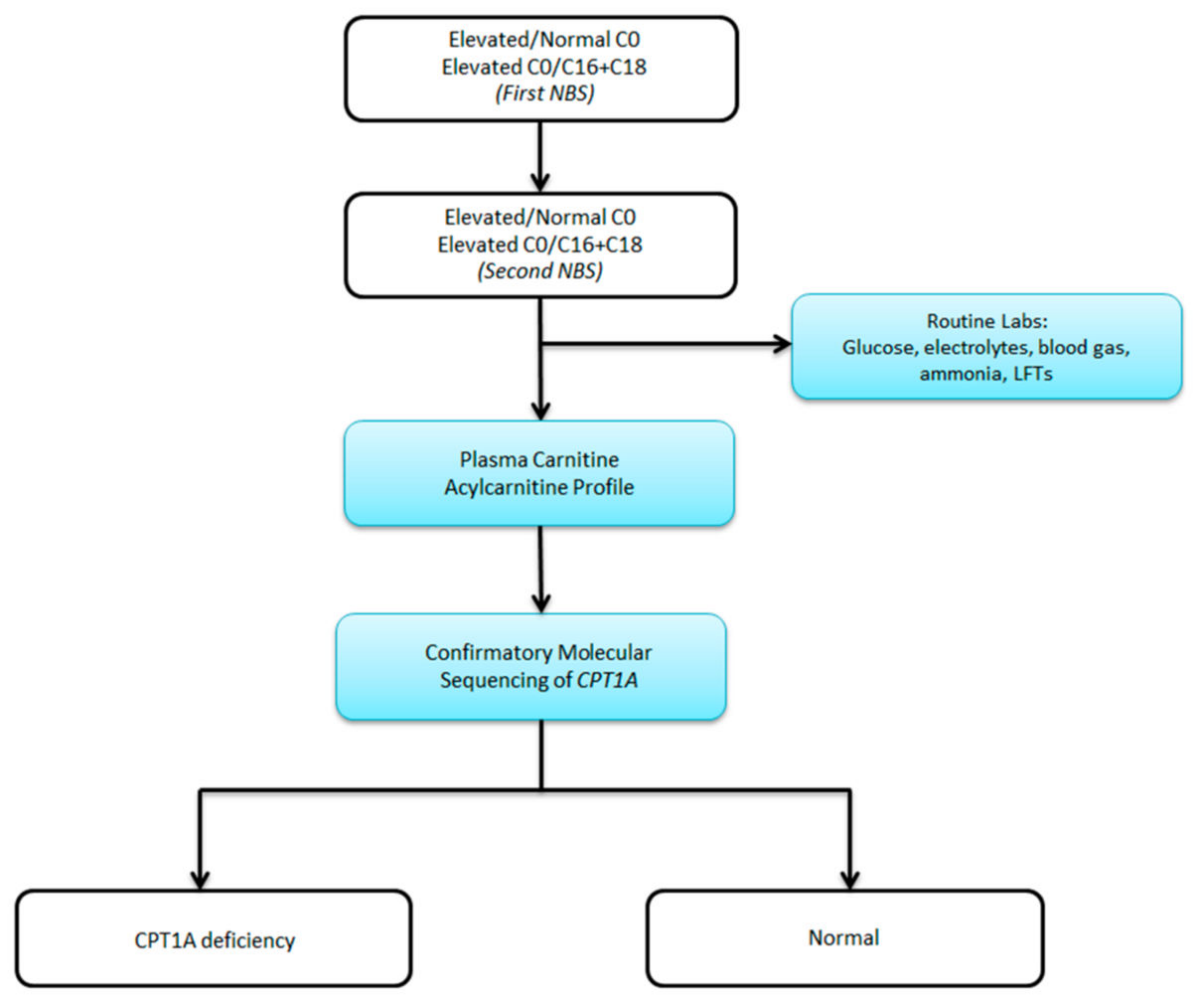
Proposed new algorithm for CPT1 deficiency on newborn screening.

Our patient had an abnormal newborn screen which was positive for CPT1 deficiency. At the time of initial follow up with a consulting metabolic physician when the patient was two weeks of age, the acylcarnitine profile and plasma carnitine levels did not suggest biochemical evidence of CPT1 deficiency. By following the NBS ACT sheet guidelines (see Figure 1), physicians were prompted to stop investigations if the confirmatory biochemical profile after the initial abnormal screen is within normal limits. There have been reports of disorders of long-chain fatty acid oxidation being missed by newborn screening [9,10]. Patients with fatty acid oxidation defects can have normal biochemical profiles in the absence of physiologic stressors. It is therefore possible to have an abnormal newborn screen but normal confirmatory metabolic testing. As a result of these missed cases, our group has adopted the practice of obtaining genetic sequencing for all abnormal newborn screens for disorders of fatty acid oxidation. Here, we propose a revised algorithm for the evaluation of positive newborn screens for CPT1 deficiency (see Figure 2).

Presenting with acute liver failure during an episode of acute viral gastroenteritis is characteristic of CPT1A deficiency. The CPT1A protein lies within the outer mitochondrial matrix and is essential for the conversion of long-chain fatty acids to acylated species so that they can be transported into the inner mitochondrial matrix for fatty acid oxidation. Molecular analysis confirmed his diagnosis of CPT1A deficiency despite only mild suggestions of biochemical disease in plasma and urine studies. While we cannot make direct correlations at the present time, it is possible that our patient had suffered neurologic sequelae due to unrecognized episodes of hypoglycemia and metabolic crises, as evidenced by his growth failure and developmental delays.

## 4. Conclusions

This case report illustrates how a patient with molecularly confirmed CPT1A deficiency was missed based on the current newborn screening ACT sheet guidelines. Although the newborn screen flagged as abnormal for the appropriate disease, the absence of biochemical evidence on subsequent metabolic testing allowed this patient to remain undiagnosed until he was 18 months of age and had evidence of metabolic decompensation. As previously reported by Wanders et al., normal acylcarnitine profiles do not rule out CPT1A deficiency and mild fatty acid oxidation defects [11]. Therefore, repeat newborn screening from a dried blood spot is a necessary step in patients with an initial positive screen for CPT1A deficiency. If one follows the current algorithm provided by the ACMG ACT sheets, normal plasma acylcarnitines lead to a “normal” outcome while only abnormal biochemical testing from plasma leads to a diagnosis of CPT1A deficiency. Our case highlights how CPT1A deficiency can be detected by abnormal newborn screening but have a normalized carnitine profile on follow up in infancy, further emphasizing the importance of a repeat dried blood spot prior to biochemical testing via plasma and enzymatic assays. Our patient also did not have confirmatory sequencing at birth, but was found to have CPT1A deficiency with molecular confirmation when he presented to our group at 18 months of age. Following the current algorithm, confirmatory CPT1A sequencing is listed only as an optional test. This current structure can lead to missed cases, which is why we propose that all patients with a positive repeat newborn screen for CPT1 deficiency have mandatory CPT1A sequencing.
